# 

**Published:** 1888-03

**Authors:** 


					﻿io cts. a copy
fJARCH; 1888.
$1.00 per year.
gJOYRNAL^f
HEALTH
ESTABLISHED 18^4
l/°±- 35
c/v — 3.
OFFICE: 206 BROADWAY, EVENING POST BUILDING, Room 11. N. Y.
Entered as Second-class Matterat the Post-Office, New York.
				

